# The Impact of Quality Improvement Measures in the Management of Asthma Patients in Juvenile Detention Facilities

**DOI:** 10.7759/cureus.13351

**Published:** 2021-02-15

**Authors:** Yvette D Toledanes, Louis Tran, Jocelyn Lara, Natali Injijian, Arianna Neeki, Fanglong Dong, Marina D Mejia Aguilar, Kylee Borger, Michael M Neeki

**Affiliations:** 1 Public Health, San Bernardino County Department of Probation, San Bernardino, USA; 2 Emergency Medicine, Arrowhead Regional Medical Center, Colton, USA; 3 Emergency Medicine, California University of Science and Medicine, Colton, USA; 4 Clinical Research, Arrowhead Regional Medical Center, Colton, USA; 5 Emergency Department, Arrowhead Regional Medical Center, Colton, USA; 6 Public Health, California University of Science and Medicine, San Bernardino, USA; 7 Emergency Medicine, San Bernardino County Department of Probation, San Bernardino, USA

**Keywords:** asthma, juvenile detention, quality improvement

## Abstract

Asthma is a serious chronic lung disease with a significant economic burden. The population of the San Bernardino County Juvenile Detention and Assessment Centers have higher odds of asthma as compared to the general population. Research has shown that a significant number of patients with a prior history of asthma were misdiagnosed. A protocol using objective testing, along with the detailed patient's history, was successfully implemented to verify the diagnosis and guide more effective medical care. After the implementation of those steps, the prevalence of asthma was found to be lower with the new protocol, from 18.1% in the pre-protocol period to 11.2% in the post-protocol period. This decrease resulted in an associated reduction in both direct and indirect healthcare costs and more efficient medical care.

## Introduction

Asthma is a chronic disease that affects nearly 25 million people or 7.7% of the total population in the United States [1]. The Centers for Disease Control and Prevention (CDC) estimated the economic burden on the United States economy at more than $80 billion per year [2]. Among young adults and children age < 18 years, more than 5.5 million, or 7.5% of that age group, reported having the disease [1]. Asthma can be life-threatening for patients if not treated properly. The American Lung Association reported that asthma is the third leading cause of hospitalization among children under the age of 15 years [3]. Despite being only 20% of the U.S. population, children of that age group represented 29% of all asthma hospital discharges in 2010 [3].

Even after adjustment for sociodemographic differences, incarcerated patients in the U.S. have higher odds of asthma and other chronic medical conditions than the general population [4]. According to the Bureau of Justice Statistics, 50% of state and federal prisoners reported having a chronic condition, with asthma being the second most commonly reported disease [5]. Wang and Green suggested that individuals with a history of incarceration were 7% more likely to have asthma as compared to their counterparts [6].

The data for the national prevalence of asthma collected by the CDC were based on patient self-reporting through a questionnaire [1]. However, one recent study suggested that asthma may be misdiagnosed in a third of these patients [7]. Consequences of these misdiagnoses include increased medication costs, potential side effects related to the unnecessary use of medications, and lost opportunities to diagnose the true cause of the patients' symptoms. Therefore, it is important to employ objective testing to confirm these self-reported asthma diagnoses.

The San Bernardino Juvenile Detention and Assessment Centers (JDAC) developed and implemented clinical protocols to improve the identification and management of patients with asthma. This study aimed to evaluate the impact of these protocols on the prevalence of asthma among the facilities’ juvenile detainees, the subsequent economic impact of treating these patients, and the compliance of healthcare providers in following these protocols.

## Materials and methods

This is a prospective observational study with retrospective chart reviews of youths aged 12 to 18 years with a self-reported history of asthma, detained at two juvenile detention facilities from July 2017 through June 2019. The two facilities were the Central Valley JDAC and the High Desert JDAC, both located in San Bernardino County (SBC), California. Data were extracted from the patients’ electronic health records (EHR). The institutional review board at Arrowhead Regional Medical Center approved this study.

SBC is the largest geographic county in the United States with a diverse population of 2.2 million people. According to the 2019 Census data, the racial makeup of SBC was 54% Latino, 27% White, 9% African American, and 8% Asian [8]. The county has a large proportion of low socioeconomic status individuals with 13.3% of the population living below the poverty level and more than a quarter of the population is younger than 18 years old [8].

Implementation of the quality improvement programs

In July 2018, the JDACs’ Continuous Quality Improvement (CQI) team developed and implemented a new clinical protocol to identify and treat patients with asthma. The protocol included an initial focused pulmonary history, the Asthma Control Test (ACT), and the use of the peak flow at each clinical encounter. Training was given to all medical services personnel, transportation officers, unit staff, and supervisors. A total of 35 educational classes with 268 participants were given at both JDACs.

All juveniles with a self-reported history or an established diagnosis of asthma were screened using a new asthma questionnaire. Where possible, the patient’s family or guardian was contacted to confirm the reported history and verify current medications and ongoing treatment regimen. The questionnaire focused on asthma-related hospitalizations and previously completed diagnostic procedures such as chest X-rays, spirometry, and other pulmonary function tests. Patients were evaluated by the medical provider within 72 hours of incarceration. Asthma diagnoses were classified according to severity (mild, moderate, or severe) and duration (intermittent or persistent). An individualized asthma action plan and follow-up schedule were developed by the medical provider. The plan of care involved educating the patient on the disease process and the effects of diet and physical activity on asthma. An accompanying chronic care checklist was created to guide the provider during their clinical encounters.

Additional safety measures were implemented at both JDACs. The “no inhalers left behind” campaign reminded staff that inhalers should follow these patients everywhere they go. Transportation officers were educated and instructed to ensure the inhalers would accompany these juveniles to their outside facility appointments. A process was developed to facilitate patients being discharged with their required inhalers and other asthma medications. In December 2018, both facilities reported 100% compliance indicating that every youth released from the JDAC went home with their rescue inhalers.

The CQI team also advocated for onsite availability of spirometry, as recommended by guidelines from the National Institute of Health (NIH) [9]. Spirometry is an important tool in diagnosing asthma and assessing the effectiveness of treatment and management of the disease. In March 2019, the in-house spirometry testing was adopted at both Central Valley JDAC and High Desert JDAC. All revisions in asthma assessment and management have been reflected in the facilities’ Standardized Protocol for Registered Nurses in early October 2019 and the Clinician’s Clinical Protocol from March 2019.

Statistical analysis

All statistical analyses were conducted using the SAS software, version 9.4 (SAS Institute, Cary, North Carolina). Descriptive statistics were presented as frequencies and percentages. Two time periods were defined: “pre-protocol”, or “pre”, is defined as the time period from July 2017 to June 2018; “post-protocol”, or “post”, is defined as the time period from July 2018 to June 2019. The asthma prevalence percentage was defined as the incidence of patients with diagnosed asthma out of the total intake population. The percentage was calculated for the overall population, as well as for both male and female subgroups. The asthma prevalence percentage was compared between the pre-protocol and post-protocol periods. The percentage was compared among the different month and year combinations through logistic regression. All statistical analyses were two-sided with a p-value < 0.05 considered statistically significant.

## Results

A total of 764 asthma patients between July 2017 and June 2019 were included in the final analysis. Five-hundred thirty-eight (538; 70.4%) patients were male and 226 (29.6%) were female. The overall patient population during this time period was 5,421, with 4,284 (81.7%) male and 967 (18.3%) female. During the pre-protocol period, the total prevalence of asthma was 463 (18.1%), with 328 (15.4%) males and 135 (31.6%) females. During the post-protocol period, the total prevalence of asthma was 301 (11.2%), with 210 (9.7%) males and 91 (16.9%) females. Table 1 presents the patient demographics.

**Table 1:** Summary of patients with asthma by month and gender *All values were presented as the number of patients with asthma divided by the number of total patients in that category.

Month	Males with asthma*	Females with asthma*	Total with asthma*	pct males w/asthma	pct females w/asthma	pct total w/asthma
Jul-17	38/169	11/33	49/202	22.5%	33.3%	24.3%
Aug-17	30/167	8/39	38/206	18.0%	20.5%	18.4%
Sep-17	28/192	13/26	41/218	14.6%	50.0%	18.8%
Oct-17	30/176	13/24	43/200	17.0%	54.2%	21.5%
Nov-17	19/167	13/24	32/191	11.4%	54.2%	16.8%
Dec-17	12/149	4/19	16/168	8.1%	21.1%	9.5%
Jan-18	20/168	4/19	24/187	11.9%	21.1%	12.8%
Feb-18	34/180	10/44	44/224	18.9%	22.7%	19.6%
Mar-18	37/197	20/54	57/251	18.8%	37.0%	22.7%
Apr-18	31/196	19/51	50/247	15.8%	37.3%	20.2%
May-18	27/200	10/50	37/250	13.5%	20.0%	14.8%
Jun-18	22/167	10/44	32/211	13.2%	22.7%	15.2%
Total pre-protocol	328/2128	135/427	463/2555	15.4%	31.6%	18.1%
Jul-18	14/145	8/47	22/192	9.7%	17.0%	11.5%
Aug-18	21/173	17/56	38/229	12.1%	30.4%	16.6%
Sep-18	23/179	11/53	34/232	12.8%	20.8%	14.7%
Oct-18	20/209	13/56	33/265	9.6%	23.2%	12.5%
Nov-18	18/179	7/48	25/227	10.1%	14.6%	11.0%
Dec-18	20/199	5/40	25/239	10.1%	12.5%	10.5%
Jan-19	23/229	2/39	25/268	10.0%	5.1%	9.3%
Feb-19	16/169	8/32	24/201	9.5%	25.0%	11.9%
Mar-19	15/169	8/43	23/212	8.9%	18.6%	10.8%
Apr-19	11/164	3/44	14/208	6.7%	6.8%	6.7%
May-19	14/166	6/46	20/212	8.4%	13.0%	9.4%
Jun-19	15/175	3/36	18/211	8.6%	8.3%	8.5%
Total post protocol	210/2156	91/540	301/2696	9.7%	16.9%	11.2%
Overall total	538/4284	226/967	764/5251	12.6%	23.4%	14.5%

**Figure 1:**
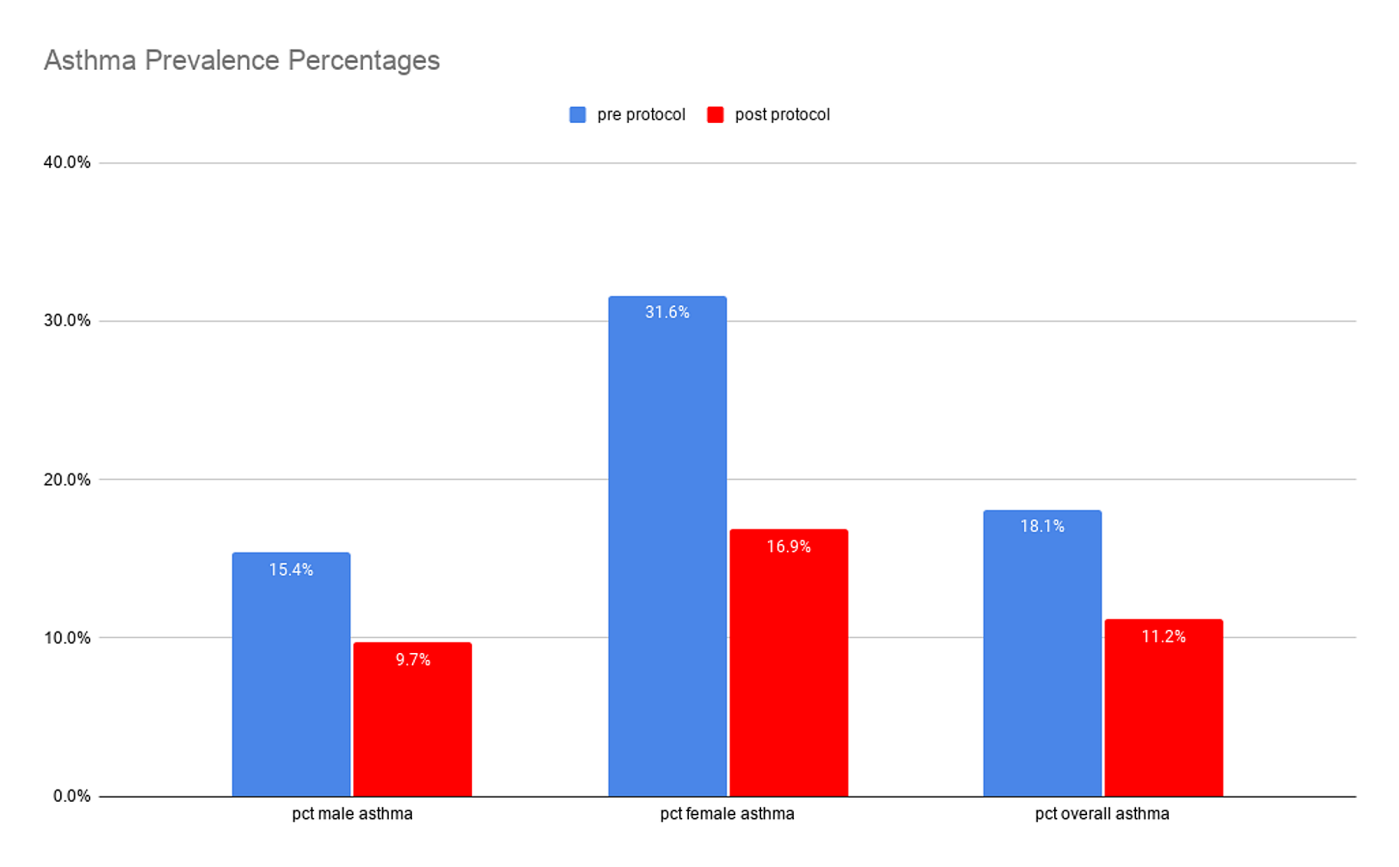
Comparison of asthma prevalence percentages in the two time periods of the study period

**Figure 2:**
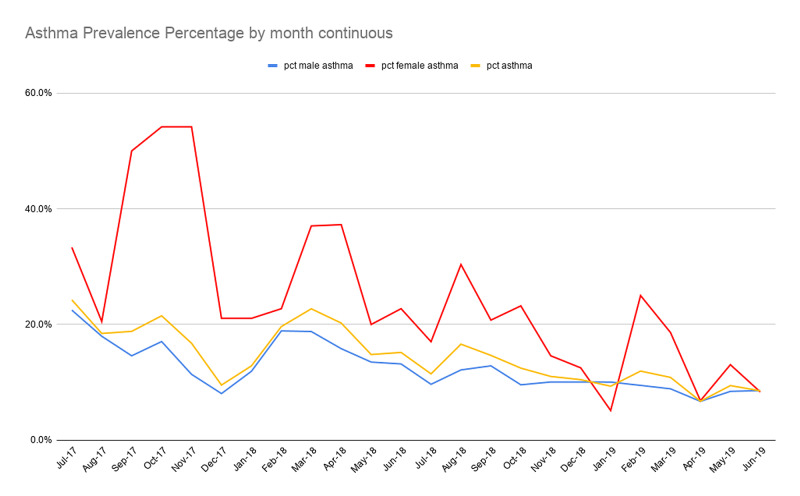
Monthly prevalence percentages of asthmatic patients

There was a statistically significant decrease in the prevalence of asthma patients in the total population between the pre-protocol and post-protocol periods (18.1% vs 11.2%, p < 0.0001). This decrease was also seen in both the male (15.4% vs 9.7%, p < 0.0001) and female (31.6% vs 16.9%, p < 0.0001) subgroups. This is represented in Figure 1. Additionally, there was a statistically significant decrease when comparing each month to the corresponding month of the pre-protocol period. The monthly decrease was seen in the overall population, as well as the male and female subgroups (all p-values < 0.0001). Figure 2 presented the decrease of patients with asthma graphically. The associated rescue inhaler cost decreased from $15,061.39 (463*$32.53) in the pre-protocol to $9,791.53 ($301*$32.53) in the post-protocol period per year, a 34.9% reduction in rescue inhaler cost, assuming that each rescue inhaler cost $32.53.

## Discussion

Asthma is a significant health problem affecting nearly 25 million people in the U.S. with an estimated economic burden of over $80 billion annually [1-2]. Children with asthma have a higher rate of annual emergency department visits, hospitalizations, outpatient visits, and prescription drug use compared to children without asthma [10-11]. In San Bernardino County, young adults and children also face the added burden of unhealthy air quality. The air pollution rate in this county consistently ranked the worst among all counties in California, affecting over 35,000 children with asthma [12]. Researchers have noted the association between exposure to air pollutants and the development and exacerbation of asthma [13-14]. This contributes to SBC having an active lifetime asthma prevalence in the highest quartile in California [15].

Additionally, the youths in this study population had a unique disadvantage. Several studies already suggested that incarcerated patients have higher odds of asthma than the general population [4-6]. Winkelman et al. investigated adolescents aged 12 to 17 years and found a higher prevalence of asthma among those who were on parole, probation, or in juvenile detention in the past year [16]. One study observed that 31.2% of incarcerated female juveniles were diagnosed with asthma [17]. However, asthma may be misdiagnosed in a significant portion of these patients, presenting a considerable challenge to the medical system of SBC JDACs [7].

To appropriately treat and manage patients with asthma, proper diagnosis is essential. Aaron et al. reported that nearly one-third of patients with physician-diagnosed asthma could not be verified when using objective testing such as peak flow, spirometry, and bronchoprovocation [7]. Several studies have demonstrated that the overall cost of misdiagnosing asthma can be substantial [2,18-19]. While healthcare cost varies by systems and locations, one study noted that the average annual cost per person was $3,266 [2]. Yaghoubi and colleagues extrapolated their findings to the US populations for a potential savings of over $56 billion over 20 years [19]. Even in smaller cohorts, such as the population of the JDACs, improper asthma diagnoses can add unnecessary burdens to the overall cost of incarceration and divert funding and resources from other essential services. According to the California Legislative Analyst’s Office, an inmate’s health care represents nearly one-third of the total cost of incarceration [20].

Implementing a diagnostic testing algorithm to verify asthma diagnosis may result in substantial savings and improvement in patients’ quality of life [19]. Additionally, an educational intervention can significantly improve patient asthma outcomes, resulting in fewer unscheduled visits and hospitalizations [21-22]. The protocol implemented by the JDACs’ CQI team was well-received, and both facilities reported full participation in the training. After 6six months, both JDACs had reported 100% compliance. Data gathered during the pre-protocol period demonstrated asthma prevalence at 18.1%, significantly higher than the national prevalence of 7.5% among children ages 0-17 years [1]. With the new clinical protocol, the prevalence dropped to 11.2% in the 12 months of the post-protocol period.

Proper identification allowed for a better allocation of resources, resulting in significant savings in both direct and indirect costs. Direct savings are seen in the decreased cost of medications and the number of medical encounters. The associated rescue inhaler cost had a 34.9% reduction in rescue inhaler cost after the implementation of this program. Correspondingly, the cost of procuring other asthma-related medicine, such as inhaled corticosteroids, was also reduced. In addition, fewer needs for asthma-related medical encounters also decreased the cost of associated personnel and transportation. Indirect savings are more difficult to assess. Patients who had been previously misdiagnosed would not be exposed to the potential side effects of unnecessary medications. Furthermore, these patients would be receiving efforts and treatment towards the true cause of their symptoms, rather than having them be falsely attributed to asthma.

Limitations

The study did not track whether a patient is a repeat encounter from a prior incarceration during the study period. This may affect the change in the prevalence, as the patient would have been correctly diagnosed from the previous visit. The cost analysis only addressed the savings from the decrease in rescue inhalers. It did not address other medicine used to treat asthma, including nebulized beta-agonists, inhaled corticosteroids, systemic steroids, or leukotriene inhibitors. It also did not address other costs, including personnel, transportation, and needless diagnostic testing. Furthermore, the study did not address the cost savings from treating the side-effects of medicines improperly given to misdiagnosed patients.

Going forward, continued surveillance and widespread educational programs for staff and patients are important to determine if the subsequent decrease in asthma prevalence in the post-protocol period is maintained. More robust data gathering should be done to thoroughly investigate the cost analysis. An emphasis on indirect cost savings should be included in future studies.

## Conclusions

The clinical protocol that included objective testing to improve identify patients with asthma in the San Bernardino JDACs was easily implemented and resulted in a statistically significant decrease in the asthma prevalence of the detained population. This resulted in better utilization of resources and the associated cost savings in providing healthcare to these patients.
